# Surface Grafted MSI-78A Antimicrobial Peptide has High Potential for Gastric Infection Management

**DOI:** 10.1038/s41598-019-53918-4

**Published:** 2019-12-03

**Authors:** Paula Parreira, Claudia Monteiro, Vanessa Graça, Joana Gomes, Sílvia Maia, Paula Gomes, Inês C. Gonçalves, M. Cristina L. Martins

**Affiliations:** 1 i3S, Instituto de Investigação e Inovação em Saúde, Universidade do Porto, Porto, Portugal; 2 INEB, Instituto de Engenharia Biomédica, Universidade do Porto, Porto, Portugal; 3 IPATIMUP, Instituto de Patologia e Imunologia Molecular, Universidade do Porto, Porto, Portugal; 4 LAQV-REQUIMTE, Departamento de Química e Bioquímica, Faculdade de Ciências, Universidade do Porto, Porto, Portugal; 5 FEUP, Faculdade de Engenharia, Universidade do Porto, Porto, Portugal; 6 ICBAS, Instituto de Ciências Biomédicas Abel Salazar, Universidade do Porto, Porto, Portugal; 7 Present Address: Universidade de Aveiro, Aveiro, Portugal; 8 Present Address: CEMUP, Centro de Materiais da Universidade do Porto, Porto, 4150-180 Portugal

**Keywords:** Microbiology techniques, Biomedical materials, Peptide delivery, Gastric cancer, Bacterial infection

## Abstract

As we approach the end of the antibiotic era, newer therapeutic options, such as antimicrobial peptides (AMPs), are in urgent demand. AMP surface grafting onto biomaterials has been described as a good strategy to overcome problems associated with their *in vivo* stability. *Helicobacter pylori* is among the bacteria that pose greatest threat to human health, being MSI-78A one of the few bactericidal AMPs against this bacterium. Here, we report that MSI-78A grafted onto model surfaces (Self-Assembled Monolayers –SAMs), in a concentration of 30.3 ± 1.2 ng/cm^2^ determined by quartz crystal microbalance with dissipation (QCM-D), was able to kill, by contact, 98% of planktonic *H. pylori* in only 2 h. This fact was not verified against the control bacteria (*Staphylococcus epidermidis*), although the minimal inhibitory concentration (MIC) of MSI-78A in solution is much lower for *S. epidermidis* (2 μg/mL) than for *H. pylori* (64 μg/mL). Our results also demonstrated that, in opposite to other bacteria, *H. pylori* cells were attracted to ethylene glycol terminated (antiadhesive) surfaces, which can explain the high bactericidal potential of grafted MSI-78A. This proof of concept study establishes the foundations for development of MSI-78A grafted nanoparticles for gastric infection management within a targeted nanomedicine concept.

## Introduction

*Helicobacter pylori* (*H. pylori*) is a Gram negative bacterium that infects more than 50% of the worldwide population^1^. This bacterium is the etiological agent of several gastro-duodenal diseases, such as chronic gastritis and peptic ulcer disease, but it is also responsible for 75% of the global gastric cancer burden^1,2^. To date, gastric cancer is the 5^th^ most common and the 3^rd^ deadliest cancer worldwide^3,4^. Consequently, *H. pylori* eradication from infected people is the best option to circumvent the infection outcomes. The available therapeutic scheme is based on the combination of at least two antibiotics plus a proton-pump inhibitor but it fails in 10–40% of the cases^5,6^. The failure of the therapeutic regimen is mostly due to the high rates of bacterial resistance to antibiotics^1,7,8^. In fact, the World Health Organization has placed *H. pylori* among the 16 antibiotic-resistant bacteria that pose the greatest threat to human health^4^. Therefore, innovative strategies based on non-antibiotic drugs are urgently required for gastric infection management.

Antimicrobial peptides (AMPs) are low molecular weight peptides that are widely distributed in living organisms as part of their immune system^9–12^. AMPs are an appealing alternative to the conventional antibiotic therapies, presenting low tendency to induce bacterial resistance, once they induce selective damage to bacterial membranes through mechanisms that bacteria find difficult to evade^11,13–17^. Up until now, more than 5,000 AMPs have been discovered or synthesized^17–19^ but only a few have been described to have anti-*H. pylori* activity, namely: Odorranain-HP^20^ and Magainin-2^21,22^. MSI-78, commercially known as Pexiganan, is a 22-amino acid peptide Magainin-2 analogue, constructed through a series of amino acid substitutions and deletions in order to make the naturally occurring Magainin-2 more active^21,22^, namely against *H. pylori*^23^. In *in vivo* settings, “unbound AMPs” can undergo proteolysis and peptide aggregation, leading to a decrease in activity^11^. An advocated strategy to bypass these drawbacks is to immobilize AMPs with anti-*H. pylori* activity, as commonly performed with other antimicrobial peptides^11^. It is thought that immobilization would confer protection against enzymatic degradation *in vivo* and prevent aggregation, therefore increasing the AMPs long-term stability, which would then enhance activity and avoid the toxicity-issues associated with the use of high AMP concentrations to achieve biological effect^11,24^. So far, MSI-78 encapsulation in nanoparticles for *H. pylori* infection treatment has been studied^25^ but no strategy concerning the AMP surface grafting onto nanoparticles for gastric infection management has been reported.

**Figure 1:**
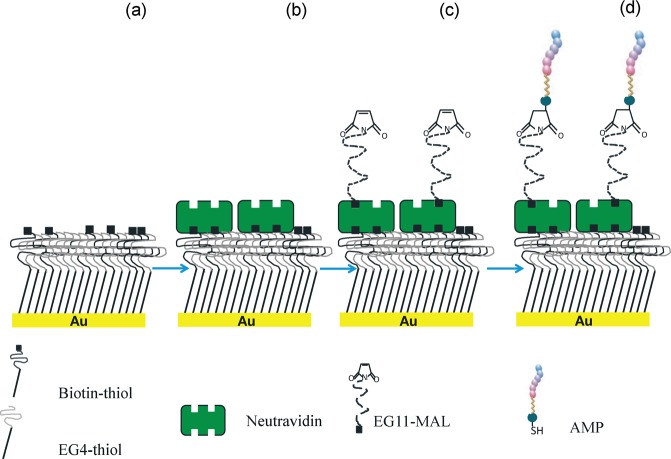
AMP immobilization onto model surfaces (biotin-SAMs). Schematic representation, not to scale. (**a**) Mixed SAMs of biotin and tetraethylene glycol (EG4)-terminated thiols (biotin-SAMs) were previously prepared and thoroughly characterized by us^28^; (**b**) Neutravidin, which strongly binds to biotin moieties protruding from the SAMs surface, was used as a protein-bridge for binding the heterobifunctional biotin-polyethyleneglycol-11-maleimide spacer (EG11-MAL); (**c**) Biotin in one end of the spacer binds to the previously immobilized neutravidin on the biotin-SAMs surface; (**d**) The maleimide group (MAL) on the opposite terminal of the spacer allows the binding of the AMP (–SH) groups (thiol-maleimide chemistry).

Planning the future development of a bioengineered non-antibiotic therapy against *H. pylori* based on surface-immobilized AMPs, this work aimed to access if, after surface-grafted, AMPs were able to retain its bioactivity against *H. pylori*. For that, model surfaces (Self-assembled monolayers - SAMs) were used in this proof of concept study. SAMs are easy to prepare, functionalize and control at molecular scale, being compatible with many surface characterization techniques used for AMP grafting detection^26,27^. Furthermore, SAMs have been previously used by us to demonstrate the specific recognition between *H. pylori* adhesins (BabA and SabA) and surface grafted glycans (Lewis b and sialyl-Lewis x)^28^, being afterwards this knowledge translated onto biocompatible polymers (chitosan microspheres)^29^. AMPs described in the literature as active against *H. pylori* were selected, namely: Odonorrain-HP^20^, MSI-78 (pexiganan)^25^ and MSI-78A^21–23^, which is derived from the MSI-78 by replacement of one amino acid (G13A). These AMPs were firstly screened in solution. Then, the most active AMP was synthesized with an extra cysteine (-SH) and with an aminohexanoic acid (ahx) spacer at either the *N-* or *C*-terminus. This modification allowed controlling both AMP orientation and exposure once grafted onto a surface. Moreover, the use of a maleimide-terminated polyethylene glycol (EG11) spacer also enhanced AMP exposure and permitted its covalent binding. The AMP immobilization strategy onto model surfaces was based on the thiol-maleimide chemistry and is described in Fig. 1.

Quartz Crystal Microbalance with Dissipation (QCM-D) was used to quantify the immobilized AMP mass on the engineered model surfaces. The *in vitro* activity of AMP-SAMs was tested using the highly pathogenic *H. pylori* J99 strain. *Staphylococcus epidermidis* ATCC 35984 strain was selected as control for bacterial adhesion onto SAMs, since its surface adhesion behaviour is well-known, namely its ability to adhere to most surfaces with exception of the non-fouling ones^30,31^.

## Results

### AMPs activity in solution

In Table 1, MIC and MBC values for the tested AMPs against the tested *H. pylori* strains are presented.

**Table 1:** AMPs antimicrobial activity against *H. pylori* J99, 26695, NTCC11637 and SS1 strains.

*H. pylori*	AMPs									
	Odorranain-HP		MSI-78		MSI-78A		HS-MSI-78A		MSI-78A-SH	
	MIC	MBC	MIC	MBC	MIC	MBC	MIC	MBC	MIC	MBC
J99	>512	>512	256	>512	64	128	16	32	64	128
26695	>512	>512	64	128	16	32	16	32	16	32
NTCC 11637	>512	>512	256	>512	128	256	128	128	128	512
SS1	>512	>512	128	>512	128	256	64	128	128	128

MIC and MBC values are expressed in μg/mL.

In our experimental settings, Odorranain-HP didn’t have antibacterial activity. MSI-78A had better antibacterial performance than MSI-78 and therefore, it underwent further modifications with an additional cysteine residue at the *N*- or *C*-terminus for later surface grafting. Of those, HS-MSI-78A had bacteriostatic (MIC) and bactericidal (MBC) effects in lower concentrations than MSI-78A-SH, being thus HS-MSI-78A the one selected for immobilization onto model surfaces (SAMs). For *S. epidermidis*, the adhesion control strain, the MIC for MSI-78A was set at 2 μg/mL.

### AMP immobilization onto model surfaces (biotin-SAMs)

**Figure 2:**
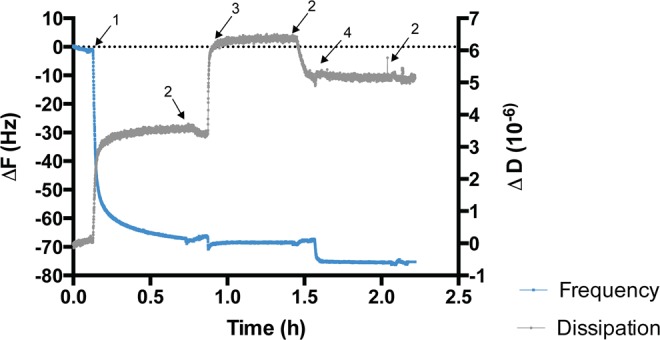
AMP immobilization onto 2.5% biotin-SAMs followed by QCM-D. ΔF- Frequency shifts; ΔD- Dissipation shifts. (1) neutravidin injection; high frequency shift correlates to large neutravidin size and high concentration. Dissipation variations also indicate protein adsorption onto 2.5% biotin-SAMs; (2) PBS rinsing; (3) EG11-MAL spacer injection; small shift in frequency is related to the PEG molecular weight and its low concentration. However, high dissipation shift indicates EG11-MAL linker incorporation onto model surface; 4) HS-MSI-78A; small shift in frequency correlates to the small size of the AMP. The decrease in the dissipation is related to AMP binding to the EG11-MAL linker, leading to a more compact (less fluid) surface.

2.5% biotin-SAMs were selected for HS-MSI-78A immobilization, since model surfaces with this biotin/EG4 ratio were previously characterized and described as the ideal one for surface functionalization with ligands (more surface immobilized mass is achieved)^28^. The QCM-D technique allowed following in real time the AMP immobilization (Fig. 2) as well as to estimate the mass of surface immobilized peptide. The frequency shifts, Δf, are related to mass changes on the crystal surface, whereas dissipation shifts, ΔD, are related to the viscoelastic properties of the adsorbed layer^32^.

After three independent experiments with duplicates, the peptide mass on the surface was calculated by applying the Sauerbrey equation to the 5^th^ overtone, which was chosen as the most stable and reproducible overtone. This equation can be applied for systems where ΔD/Δf is lower than 4 × 10^–6^ Hz^−1^ and assumes that the adsorbed/bound film is rigid with no internal loss of energy, which is translated in low values of dissipation^33^. The immobilized mass of HS-MSI-78A was estimated to be 30.3 ± 1.2 ng/cm^2^, but this may be an overestimation, since this value may be taking into account water and other buffer constituents trapped between the adsorbed molecules^34^.

### Antibacterial activity of the AMP-functionalized model surfaces (AMP-SAMs)

**Figure 3:**
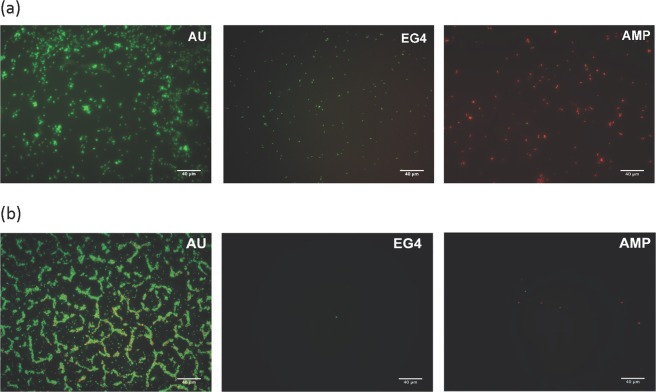
Representative images of (**a**) *H. pylori* J99 and (**b**) *S. epidermidis* ATCC 35985 labelled with Live/Dead staining adhered to the different surfaces tested. Images were collected using an inverted fluorescent microscope with 400x magnification. Scale bar: 40 μm.

The antibacterial performance of AMP-SAMs (SAMs with the antimicrobial peptide HS-MSI-78A immobilized on its surface) was tested against the *H. pylori* J99 strain, a highly pathogenic human strain that is associated with poor patient prognosis^35^. AMP-SAMs activity was also evaluated against *S. epidermidis* ATCC 35984 strain, the control for surface adhesion behaviour. The viability of planktonic cells after exposure to the bioengineered model surfaces was evaluated by performing CFU counting. On the other hand, for surface adherent cells, a Live/Dead staining was used, which allowed distinguishing viable and non-viable bacterial cells (Fig. 3).

**Figure 4:**
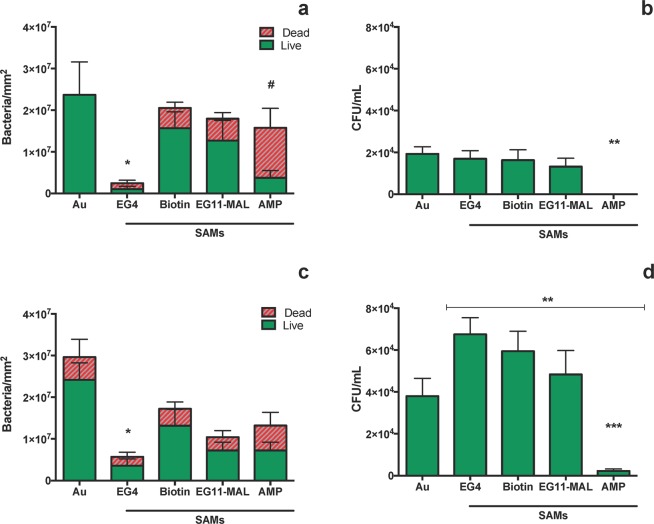
Antimicrobial activity of AMP-SAMs against *H. pylori* J99 after: 2 h incubation in PBS (**a**) surface adherent cells (**b**) planktonic cells; 6 h incubation in recovery medium (**c**) surface adherent cells (**d**) planktonic cells. Results are expressed as average ± SD. *- Total adhesion (number of live + dead cells) in EG4-SAMs significantly different from all the other tested surfaces (p < 0.05); #- number of dead bacteria significantly different from live bacteria (p < 0.05); **/***-significantly different from Au (**p < 0.001; ***p < 0.001).

Figure 4 highlights the antibacterial performance of AMP-SAMs and control surfaces (Au, EG4, Biotin, EG11-MAL) against *H. pylori* J99.

The 2 h adhesion was chosen based on previous assays, where it was determined that bacterial adhesion onto these model surfaces plateaus after 2 h^27,36^. No significant differences (p < 0.05) were observed for global adhesion (live and dead bacteria) among the tested surfaces, except for the EG4-SAMs, where adhesion was much lower in comparison to the other surfaces and also in accordance with prior findings (Fig. 4a)^27^. After 2 h, the majority of *H. pylori* adhered to control surfaces (Au, EG4, biotin and EG11-MAL) remained viable, in contrast to the 75% of non-viable *H. pylori* adhered to the AMP-SAMs (Fig. 4a). Regarding planktonic cells, no live bacteria were seen in the supernatants after 2 h of incubation with AMP-SAMs, but *H. pylori* viability was kept in control surfaces (Au, EG4, Biotin, EG11-MAL) (Fig. 4b). To further assess the antibacterial effect, namely to understand if surface-adherent bacteria were able to recover from exposure to AMP-SAMs, a set of surfaces was subsequently incubated in recovery media (MHB + 10%FBS) for 6 h (Fig. 4c). The incubation period for this recovery assay was based on the estimated duplication time for *H. pylori* (6 h)^37^. After 6 h in culture medium (Fig. 4c) and compared with 2 h (Fig. 4a), no significant increase was observed for overall adhesion (total number of live and dead bacteria). However, the number of viable and cultivable bacteria in the supernatants increased for all control surfaces (Fig. 4d). This increase may be translated as adherent live bacteria that were able to detach from the surfaces and proliferate in solution. More importantly, only a very small number of live bacteria that might be adhered to AMP-SAMs were released and able to thrive (Fig. 4d). Although MHB is the standard culture media for MICs determination in accordance with the CLSI guidelines, it is not the optimal recovery media for *H. pylori*, since this is a fastidous microrganism that requires nutrient enriched culture media^38^. To clarify if this could be interfering with the bacterial recovery rate, identical assays were performed using Brucella Broth culture medium. Similar results were obtained using both culture media, demonstrating that the use of MHB was not affecting bacterial recovery (data not shown).

**Figure 5:**
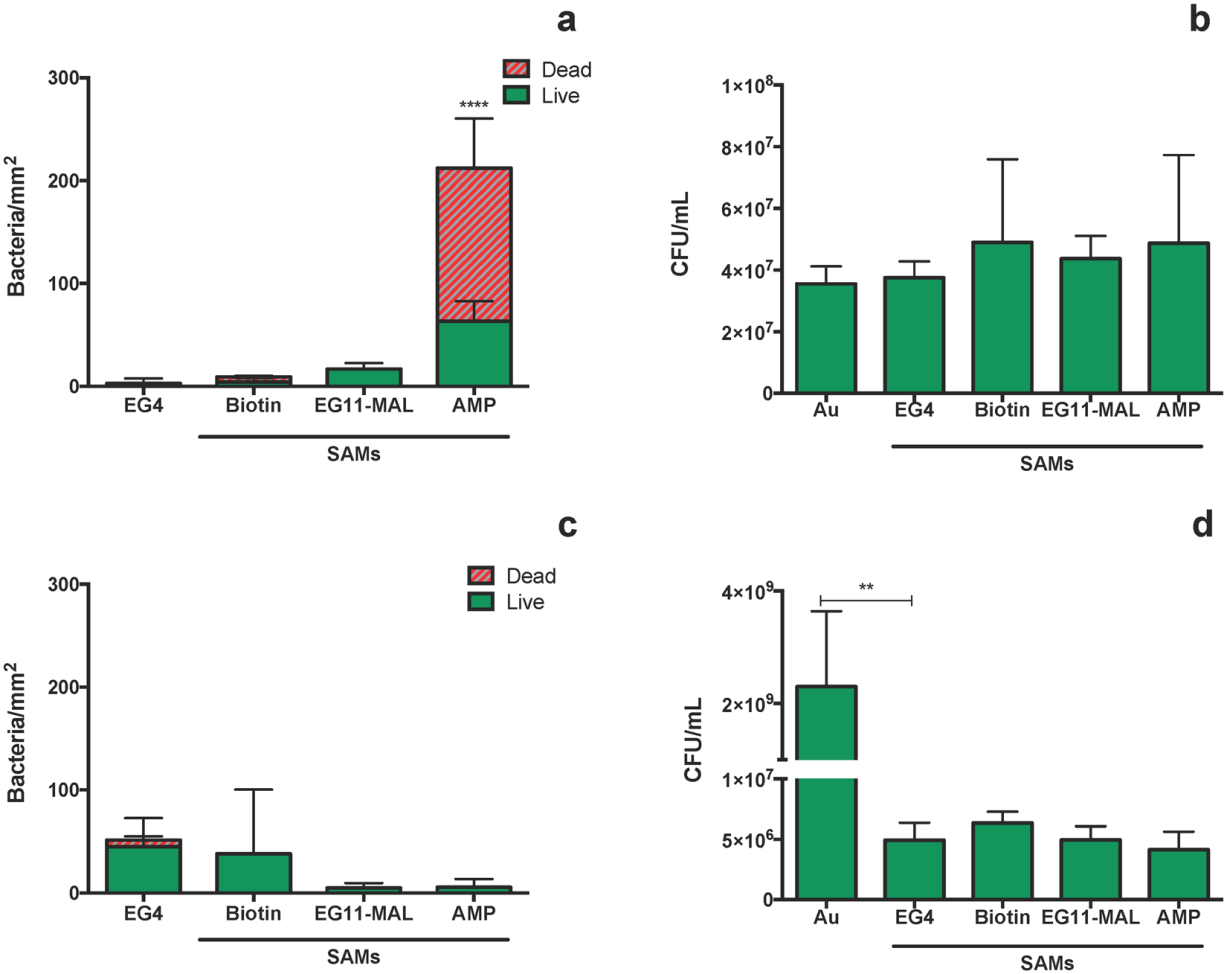
Antimicrobial activity of AMP-SAMs against *S. epidermidis* cells after: 2 h incubation in PBS (**a**) surface adherent cells (**b**) planktonic cells; after 4 h incubation in recovery medium (**c**) surface adherent cells (**d**) planktonic cells. Results are expressed as average ± SD. ** significantly different from Au (p < 0.0001); **** number of dead bacteria significantly different from live bacteria (p = 0.0013).

It is noteworthy that, although in lower number, *H. pylori* cells still adhered to EG4-SAMs, which are described as “anti-adhesive” surfaces (Fig. 4a,c). To verify the non-fouling properties of the EG4-SAMs, and that there is no AMP release, further studies were performed using the *S. epidermidis* ATCC 35984 strain and results are represented in Fig. 5.

After 2 h of incubation*, S. epidermidis* did not adhere to EG4-SAMs, while AMP-SAMs promoted adhesion and subsequent killing of adhered bacteria (Fig. 5a). Yet, the number of total surface-adherent bacteria was remarkably lower than those observed for *H. pylori* J99 (Fig. 4a). After 2 h, the number of planktonic *S. epidermidis* cells was not affected by exposure to AMP-SAMs, with no significant differences being observed between AMP-SAMs and control surfaces (Fig. 5b). After 4 h in fresh culture media, there were no significant differences among the tested model surfaces, which is in agreement with the number of live bacteria after 2 h of adhesion, also similar between samples (Fig. 5c). After 4 h in fresh culture media (TSB), more *S. epidermidis* planktonic cells were observed in all samples, including AMP-SAMs (Fig. 5d). For both incubation times, high *S. epidermidis* adhesion was observed on gold, which made the quantification of adherent bacteria unfeasible.

## Discussion

This work reports the proof of concept studies for the future development of bioengineered nanoparticles with surface grafted MSI-78A peptide as an antibiotic-free therapy against *H. pylori*.

First, different antimicrobial peptides previously reported as having anti-*H. pylori* effect were screened in solution. However, Odorranain-HP didn’t present antibacterial activity against the tested *H. pylori* strains panel (Table 1). This diverges from the previous work of Chen *et al*., where Odorranain-HP presented a 20 μg/mL MIC against *H. pylori* NTCC11637 strain^20^. A possible explanation might reside in the different experimental settings, namely the use of a different culture media. The remaining tested AMPs were active against *H. pylori* (Table 1). Of all, MSI-78A was the most effective. Therefore, the antimicrobial peptide MSI-78A was synthesized with an aminohexanoic acid (ahx) spacer and an extra cysteine (-SH) at the *N-* or *C*-terminus, envisioning its later surface immobilization in a controlled manner. Once the HS-MSI-78A demonstrated improved activity in solution (Table 1), it was selected for surface immobilization.

A key feature concerning the surface immobilization of the selected AMP is to retain its bioactivity, i.e., keeping its ability to bind to the desired target and maintaining minimum interactions with non-relevant matrix or assay components. To accomplish this, it is common to use highly hydrophilic PEG linkers, hence creating a microenvironment that decreases non-specific binding and provides additional mobility to the tethered AMPs^39^. Although the selected peptide was synthesized with a small ahx spacer, it was also used an EG11-unit spacer (EG11-MAL) for HS-MSI-78A immobilization. The use of this longer PEG allows to decrease peptide aggregation and subsequently boost the AMP binding, since it imparts water solubility that is transferred to the biotinylated molecule, ultimately improving AMP exposure from the surface^39^. A smaller EG-spacer (EG2-unit) was also tested but according to the performed QCM-D assays it demonstrated to be less efficient than the EG11-unit spacer, with lower mass of AMP immobilized on the surface (data not shown). In addition, to further justify the EG11-unit choice, it has been reported that antimicrobial peptides immobilized via EG spacers with longer arms (EGn; n > 8) yield higher antimicrobial activity than those tethered via shorter EG linkers^40^. The successful AMP immobilization onto nanostructured model surfaces (SAMs) was confirmed by the QCM-D assays (Fig. 2).

Then, the antibacterial activity of the engineered model surfaces functionalized with the selected AMP (AMP-SAMs) was evaluated. CFU counting assessed the viability of planktonic cells, while viability of surface adherent cells was evaluated using the Live/Dead Baclight Kit^TM^. This dual staining kit allows to quantify and distinguish live from dead cells. It is composed of two fluorophores, SYTO9 and propidium iodide (PI), and is based on detection of membrane integrity: cells with a compromised membrane are considered dead or dying and will stain red (PI); whereas cells with an intact membrane will stain green (SYTO9). It is important to highlight that some bacterial cells incubated with SAMs stained yellow (Fig. 3). This may occur when SYTO9, which can be actively exported from the cytoplasm, is not completely replaced by PI, generating the yellow staining^41^. As SYTO9 and PI binding and releasing to/from nucleic acids are dynamic processes, it is possible that both green and red dyes were simultaneously retained within cells, originating the yellow staining. Since this indicates cells with damaged membrane, the yellow-stained bacteria were considered as dead cells^41^.

Regarding the antibacterial activity, after 2 h most of the *H. pylori* cells adhered to control surfaces (Au, EG4, biotin and EG11-MAL) were viable, in contrast to the 75% of non-viable *H. pylori* on AMP-SAMs surface (Fig. 4a). The excellent bactericidal effect of the AMP-SAMs was further demonstrated since after 2 h of incubation there were no live planktonic bacteria detected, while *H. pylori* viability was kept in the control surfaces (Au, EG4, Biotin, EG11-MAL) (Fig. 4b). After 6 h in culture medium (Fig. 4c) and compared with 2 h (Fig. 4a), there was no significant increase observed for the overall adhesion (total number of live and dead bacteria). This was not expected because 6 h is the estimated duplication time for *H. pylori*^27^. But since bacteria are coming from a potential stressful situation, namely incubation in PBS and exposure to abiotic model surfaces (SAMs), this might result in a slower growth/duplication rate, which could also explain some of the dead bacteria observed on the gold surface after 6 h. The AMP is covalently immobilized onto SAMs surface via the cysteine-maleimide reaction that ocurrs between the AMP and the EG11-spacer and thus, it is not likely that leaching from the surface occurs. Therefore, we hypothesize that AMP-SAMs have a contact-killing effect: first, *H. pylori* cells are attracted to the AMP-SAMs, interact with the exposed AMP and are killed; then, the non-viable bacteria are released from the surface, leaving the surface immobilized AMP available to interact with/kill other *H. pylori* cells. Dead bacteria must be released from the surface in order to obtain the herein reported high death rates. The assumption that the AMP is probably not being actively leached from the surface is further encouraged by the results obtatined with *S. epidermidis*: after 2 h of incubation, *S. epidermidis* planktonic cells were not affected by exposure to AMP-SAMs with no significant differences reported between AMP-SAMs and the control surfaces (Fig. 5b). Also, the number of planktonic *S. epidermidis* cells kept increasing even after 4 h incubation with AMP-SAMs (Fig. 5d).

Furthermore, *H. pylori* cells were not able to recover from exposure to AMP-SAMs, since after 6 h in recovery culture medium only a small number of the live bacteria that adhered to AMP-SAMs (Fig. 4b) were able to detach from the surface and thrive in solution (Fig. 4d). On the other hand, a higher number of bacteria in supernatants were observed for control surfaces (Fig. 4d), indicating that in more favourable conditions bacteria can easily detach from the surface and grow. This might also explain why EG4-SAMs, which had fewer *H. pylori* cells adhered at 2 h (Fig. 4a), generated a similar number of planktonic bacteria compared to the other surfaces (Fig. 4d) when transferred to culture medium.

EG4-terminated surfaces are described as non-fouling surfaces, i.e., resistant to cell and protein adhesion^25,40,42^. Although *H. pylori* adhesion to EG4-SAMs was much lower when compared with the other model surfaces (Fig. 4a,c), values were higher than expected. Therefore, *S. epidermidis*, whose low adhesive behavior to hydrophylic surfaces is well documented, was selected as adhesion control, while also enabled to establish the non-fouling nature of EG4-SAMs^42,43^. As anticipated, very few *S. epidermidis* cells adhered to EG4-SAMs (Fig. 5a). Moreover, the low number of *S. epidermidis* adhered to the control surfaces, with gold being the exception, may be related to the common underlying EG-nature of those SAMs (Fig. 5a,c). Overall, this highlights a distinct *H. pylori* adhesion behaviour towards EG-based SAMs, which might be related with *H. pylori* proficiency in binding to the highly hydrophilic gastric mucus layer^44^.

It was also observed a considerable difference between *H. pylori* adhesion to 2.5% biotin-SAMs (composed of 97.5% EG4-thiol) and “pure” EG4-SAMs (100% EG4-thiol). Biotin has been proposed to be a critical and limited nutrient during infections by human pathogens such as *Mycobacterium tuberculosis*^45^. Nonetheless, *H. pylori* is able to produce biotin, as biotin supplementation has no effect on its growth in a chemically defined media^45^. Also, analyses of the available *H. pylori* genomes showed that it encodes all the proteins required for assembly of the fused heterocyclic rings of biotin^46,47^. However, as biotin is a key protein-bound enzyme cofactor that plays essential roles in the transfer of CO_2_ in key central metabolic processes^46^, we hypothesize that the biotin moieties readily available on the surface of the bioengineered models may act as chemo attractant. This would justify the marked difference between *H. pylori* adhesion onto EG4-SAMs and 2.5% biotin-SAMs.

Theoretically, both encapsulation and immobilization processes are able to circumvent AMPs potential limitations, such as short half-life (proteolytic degradation). In fact, nanoparticles loaded with Pexiganan (MSI-78) were recently evaluated against *H. pylori* with promising *in vitro* and *in vivo* results^25^. Nonetheless, and although surface immobilization of AMPs has been widely studied for other purposes, such as hindering biofilm formation, very few work has been devoted to the immobilization of AMPs with anti-*H. pylori* activity within gastric infection scenario. Differently from encapsulation strategies, the surface immobilization of AMPs allows to control the peptide orientation, concentration and exposure, which may play a crucial role in boosting the performance of AMP-based strategies. Also, immobilization processes enable the use of lower AMP concentrations to achieve biological effect, since it is not dependent upon encapsulation processes that may result in low encapsulation yield and may diminish the possibility of toxicity-related issues. Moreover, functionalized surfaces with covalently bound AMP are not required to undergo any other event, such as material/composite degradation for peptide release and the chance of peptide aggregation is much lower during immobilization than when performing encapsulation. Another important aspect is how immobilization may tailor and improve the AMP specificity towards the gastric pathogen. The unbound AMP was 32 times more active in solution against *S. epidermidis* than against *H. pylori*, but once surface immobilized, higher selectivity towards *H. pylori* was achieved, being the effect of the AMP-functionalized surfaces against *S. epidermidis* residual.

The study herein reported demonstrates that the bioactivity of the peptide is retained after surface immobilization. In addition, the high bacterial eradication rates obtained after 2 h (>90%) using the surface grafted MSI-78A is a very promising result, once framed with the time of stomach emptying and the effective retention time of a possible oral strategy against *H. pylori*. This proof of concept study establishes the promising potential of immobilized AMPs as non-antibiotic alternatives to counteract *H. pylori* infection, encouraging translation of the obtained knowledge onto “real-world” bioengineered approaches.

## Conclusion

This study demonstrates that not only MSI-78A can be surface-grafted without compromising its activity but also its immobilization onto non-adhesive surfaces boosts the antibacterial performance against *H. pylori*. Overall, the MSI-78A-functionalized model surfaces were highly effective against *H. pylori*. The gastric pathogen was killed in a short time span, since after 2 h only 2% of *H. pylori* cells remained viable in suspension. Furthermore, we also established that AMP-SAMs have higher activity towards *H. pylori* than to *S. epidermidis*. In summary, the herein reported proof-of-concept study validates the development of a bioengineered approach based on surface-grafted antimicrobial peptides aiming *H. pylori* eradication via non-antibiotic options.

## Methods

### Antimicrobial peptides (AMPs)

Tested AMPs and their respective sequences are listed in Table 2.

**Table 2:** AMPs and their sequences.

AMP	Peptide sequence
Odorranain-HP MSI-78 (Pexiganan)	GLLRASSVWGRKYYVDLAGCAKA GIGKFLKKAKKFGKAFVKILKK
MSI-78A (PexigananA)	GIGKFLKKAKKF**A**KAFVKILKK
HS-MSI-78A (HS-PexigananA) MSI-78A-SH (PexigananA-SH)	**HS-ahx**-GIGKFLKKAKKF**A**KAFVKILKK GIGKFLKKAKKF**A**KAFVKILKK**-ahx-SH**

AMPs synthesis was performed as previously described^13^. The last two rows on Table 2 refer to MSI-78A derivatives on which an additional cysteine residue (represented by -SH instead of its standard single-letter code C, in order to emphasize its role as a thiol donor) was attached at either the *N*- or *C*-terminus of the bioactive sequence, through a 6-amino-hexanoic acid (ahx) spacer. Afterwards, AMPs were freeze dried and stored at −20 °C. Prior to use, AMPs were suspended in phosphate-buffered saline (PBS; pH~ 7.5) to a final 1 mg/mL concentration.

### Bacteria

#### *Helicobacterpylori*

*H. pylori* human strains J99 and 26695 (provided by Department of Medical Biochemistry and Biophysics, Umeå University, Sweden), NCTC 11637 (ATCC 43504) and mouse-adapted SS1 strain (provided by Unité de Pathogenèse de *Helicobacter*, Institut Pasteur, France) were grown following the standard procedure. Briefly, *H. pylori* was cultured in Blood Agar (BA; Oxoid) supplemented with 10% of defibrinated horse blood (Probiologica) and with an antibiotic cocktail (polymyxin B, vancomycin, amphotericin B, trimethoprim; all from Sigma-Aldrich) under microaerophilic conditions (<5% O_2_; GenBox System, BioMérieux) at 37 °C for 48 h. Afterwards, some colonies were streaked onto fresh BA medium and kept in culture in the same conditions for another 48 h. Subsequently, bacterial inoculum was adjusted to 0.03 optical density at 600 nm (OD_600_), which corresponds to approximately 1 × 10^7^ colony forming units (CFUs)/mL^48^. Initial inoculum was confirmed by inoculation of 10 μL drops of the bacterial suspension onto BA and CFUs were counted after 5 days of incubation in the same above-mentioned settings.

#### *Staphylococcusepidermidis*

*S. epidermidis* ATCC 35984 strain was selected as the control for bacterial adhesion onto SAMs. This choice was based on the fact that, although being remarkbly good at adhere, proliferate and establish biofilms, this strain is not capable of adherence to non-fouling surfaces^30,31^. Bacteria were grown on Trypticase Soy Agar (TSA; Merck Millipore) plates overnight at 37 °C (spreading) and then transferred to Trypticase Soy Broth (TSB; Merck Millipore). Incubation proceeded overnight at 37 °C, 150 rpm. Bacterial inoculum was then adjusted at OD_600_ to 0.33, which corresponds to approximately 1 × 10^8^ CFUs/mL in TSB. This was optimized in order to have adherent cells onto model surfaces. The initial inoculum was confirmed by growing 10 μL drops of the suspension in TSA plates during 24 h at 37 °C.

### AMPs activity in solution

#### Minimal Inhibitory concentration (MIC)

The MICs of the AMPs (Table 1) against *H. pylori* strains were determined using the microbroth dilution method in Mueller Hinton Broth (MHB) supplemented with 10% of inactivated Fetal Bovine Serum (FBS; Gibco) and in accordance with the Clinical Laboratory Standards Institute (CLSI) guidelines^49^. Briefly, AMPs were diluted in 0.01% of acetic acid with 0.2% Bovine Serum Albumin (BSA; Sigma) to allow AMP protonation and avoid its aggregation, respectively, following an already established protocol^13^. The selected AMPs concentrations ranged from 2 µg/mL up to 512 µg/mL. Incubation was performed at 37 °C in microaerophilic conditions for 72 h. The MIC was defined as the first AMP concentration that prevented visible (naked eye) bacterial growth. Two replicates of each AMP concentration were performed in three independent experiments.

#### Minimal bactericidal concentration (MBC)

The MBC was defined as the lowest AMP concentration that was able to diminish the viability of the initial bacterial inoculum by ≥99.9%. MBC determination followed the CLSI guidelines^49^ but briefly, a 50 µL sample was taken from the first three lowest concentrations where no bacterial growth was observed (concentration corresponding to the MIC), serially diluted in PBS until 10^−6^ and then spotted on BA. After 5 days of incubation at 37 °C in microaerophilic environment, the CFUs/mL were calculated. Two replicates of each AMP concentration were performed in three independent experiments.

#### AMP-functionalized model surfaces

AMP immobilization strategy is described in Fig. 1.

#### Gold substrates

Gold substrates (1 × 1 cm^2^) used in bacterial assays were obtained from *Instituto de Engenharia de Sistemas e Computadores – Microsistemas e Nanotecnologias*, Portugal (INESC-MN)^50^. For Quartz Crystal Microbalance with Dissipation (QCM-D) assays, gold-coated quartz crystal sensors (QSX301-Standard Gold, 4.95 Hz, 78 mm^2^ active sensor area) were obtained from Biolin Scientific.

#### Model surfaces: Biotin Self-Assembled Monolayers (biotin-SAMs)

1-Mercapto-11-undecyl tetra (ethylene glycol) (SH-(CH_2_)_11_-O-(CH_2_-CH_2_-O)_4_-H; EG4-thiol; 99%, SensoPath Technologies) and biotin-terminated tri(ethylene glycol) undecanethiol (SH-(CH_2_)_10_-CO-NH-(CH_2_)_3_-O-(CH_2_CH_2_O)_2_-(CH_2_)_3_-NH-Biotin; biotin-EG3-thiol, 99%, SensoPath Technologies) were prepared as pure solutions at 2 mM in absolute ethanol (Merck). Biotin-SAMs were prepared by immersing the gold coated surfaces (gold substrates or QCM-D crystals) in solutions containing 2.5% biotin-thiol (97.5% EG4-thiol) with 0.1 mM final concentration, as previously described by us (Fig. 1a)^28^. The whole process was performed under a dry nitrogen atmosphere inside a glove box. Incubation was performed at RT (~25 °C) during 20 h. After incubation, biotin-SAMs were rinsed with absolute ethanol, dried with a gentle nitrogen stream and used immediately.

#### AMP immobilization onto model surfaces (SAMs)

AMP immobilization onto model surfaces (biotin-SAMs) was followed in real time using a QCM-D device (Q-Sense E4 system, Biolin Scientific) at RT (~25 °C). Biotin-SAMs assembled onto QCM-D gold-coated crystals (section 2.4.1 and 2.4.2) were placed in the system. Afterwards, filtered PBS (0.22 μm pore size) was injected until a stable signal was obtained (baseline). Then, neutravidin (Invitrogen), 1 mg/mL in PBS, was incubated in static for 1 h (Fig. 1b). This neutravidin concentration allowed blocking all biotin moieties freely available on the biotin-SAMs surface, as previously determined by us. Next, rinsing was done with PBS followed by a 30 min static incubation of EZ-link^TM^ maleimide (MAL)-polyethylene glycol (EG11)-Biotin spacer (EG11-MAL; Thermo Fisher Scientific) (Fig. 1c) in a concentration of 1 mg/mL in PBS. The terminal MAL group of the heterobifunctional spacer reacts specifically and efficiently with sulfhydryl groups (-SH) of the added cysteine to the AMP establishing stable covalent bonds. After, rinsing was performed with PBS and the selected AMP (HS-MSI-78A) at 0.5 mg/mL in PBS was incubated for 45 min. Then, a final rinsing with PBS was done to remove unbound peptide (Fig. 1d). All solutions were injected at flow rate of 0.1 mL/min.

Once the changes in the system dissipation were considered low (<4), the mass of the surface immobilized AMP was estimated using the Sauerbrey Eq. (1) after 4 independent experiments and expressed in ng/cm^2^.

1 $$\Delta m=-\,(\Delta f{(}\mathrm{Hz}{/}n).{\rm{C}}$$

(1) Δm is the adsorbed mass, Δf is the frequency shift due to the adsorption, n is the overtone number and C is a constant characteristic of the sensor crystal (C = 17.7 ng·Hz^−1^·cm^−2^ for the 5 MHz crystals used).

*In vitro* antibacterial activity assays were performed with model surfaces (biotin-SAMs) functionalized with the HS-MSI-78A peptide (AMP-SAMs), following the incubation settings defined in the QCM-D experiments (based on 2.3 section results).

#### Antibacterial activity of the AMP-functionalized model surfaces (AMP-SAMs)

The antibacterial activity of AMP-functionalized surfaces (AMP-SAMs) was tested against *H. pylori* J99 strain (highly pathogenic and human strain) and *S. epidermidis* ATCC 35984 strain (control for bacterial adhesion onto surfaces). Bare gold surfaces (Au), EG4-SAMs (EG4), biotin-SAMs (Biotin) and biotin-SAMs + neutravidin + biotin-EG11-MAL linker (EG11-MAL) were used as surface controls.

*H. pylori* J99 and *S. epidermidis* ATCC 35984 were simultaneously incubated with surfaces (AMP-SAMs and controls) at 37 °C and 150 rpm under microaerophilic conditions. After 2 h of incubation, surfaces were divided into two groups. From the first group of surfaces, supernatants (planktonic cells) were removed, serially diluted and platted either in BA (*H. pylori*) or TSA (*S. epidermidis*) for CFU determination. *H. pylori* CFUs were determined after 5 days of incubation at 37 °C in microaerophilic conditions, while *S. epidermidis* CFUs counting were performed after 24 h of incubation at 37 °C. After supernatant removal, surfaces were rinsed with PBS and stained with Live/Dead Baclight Kit^TM^ (Invitrogen) according to the manufacturer’s protocol. Bacteria were visualized with an Inverted Fluorescence Microscope (Zeiss Axiovert 200 MOT) at 400x magnification. Bacterial quantification was manually determined from 15 photographs (5 random fields per sample) and expressed as bacteria/mm^2^. Aiming to determine if bacteria were able to recover from exposure to AMP-SAMs, the second group of surfaces was rinsed with PBS and transferred to MHB + 10%FBS (*H. pylori*) for more 6 h (8 h incubation total) or to TSB (*S. epidermidis)* for additional 4 h (6 h incubation total) (the selected incubation times were based on the growth kinetics of each bacteria). After the mentioned incubation periods, supernatants (planktonic cells) and surfaces were processed as mentioned for the 1^st^ group. Three independent experiments were performed using samples in triplicate.

### Statistical analysis

Statistical analysis was done with GraphPad Prism software (GraphPad Software Inc., version 6.0). Data was analysed by D’Agostino & Pearson omnibus normality test. Two-way ANOVA was used to determine statistical significance in the assays concerning surface adherent cells, followed by Dunnett’s multiple comparison tests. One-way ANOVA was used for statistical analysis of the assays regarding planktonic cells, followed by a Sidak’s multiple comparison test. A value of p < 0.05 was considered statistically significant (*p < 0.05; **p < 0.01; ***p < 0.001; ****p < 0.0001).

## Data Availability

The datasets generated during and/or analysed during the current study are available from the corresponding author on reasonable request.
